# Facial Atopic Dermatitis Treated with Chitosan

**DOI:** 10.3390/life16101657

**Published:** 2026-10-02

**Authors:** Kyu-Ho Yi, Han Earl Lee, Hongseok Kim, Wonseok Choi, Jin Hyun Kim

**Affiliations:** 1 You and I Clinic, Seoul 06123, Republic of Korea; kyuho90@daum.net; 2 Opening Plastic Surgery Clinic, Seoul 06038, Republic of Korea; 3 VOS Dermatology Clinic, Gangnam-gu, Seoul 06022, Republic of Korea; 4 V Plastic Surgery, Daegu 41940, Republic of Korea

**Keywords:** atopic dermatitis, facial eczema, chitosan, barrier repair, moisturization, *Staphylococcus aureus*, photography, global improvement scale

## Abstract

**Background/Objectives:** Facial atopic dermatitis (AD) is burdensome, and facial skin is vulnerable to steroid-related adverse effects. This study describes the clinical use, tolerability, and photographic response to topical 2% chitosan gel in a consecutive case series. **Methods:** Twenty patients applied the gel to affected facial areas twice daily for 4 weeks alongside standardized gentle skin care. Clinical and patient-reported outcomes were collected, and two independent physicians assessed paired photographs using the Photographic Atopic Improvement Scale (PAIS). An additional ultrasound-based imaging record was considered separately from the cohort analysis. **Results:** The chitosan-containing regimen was associated with improvement in overall facial appearance and the available clinical and patient-reported outcomes. Photographic responses varied, with residual inflammatory lesions in some examples. Mild transient stinging occurred in 3 of 20 patients (15%) and resolved spontaneously; no serious adverse events were recorded. The additional imaging record was descriptive and did not provide an independently validated measure of skin-quality improvement. **Conclusions:** Topical 2% chitosan gel was feasible and generally well tolerated in this hypothesis-generating series. The uncontrolled design, concomitant moisturization, and rescue treatment in two patients preclude attribution of improvement solely to chitosan. Controlled studies are needed to establish efficacy and validate the photographic and device-based outcomes.

## 1. Introduction

Atopic dermatitis (AD) is a chronic, relapsing inflammatory dermatosis characterized by pruritus, eczematous lesions, and a fluctuating course. It results from a multifactorial interplay of epidermal barrier dysfunction, immune dysregulation with type 2 skewing, neuroimmune itch pathways, and microbial dysbiosis [1,2]. Across age groups, AD is associated with impaired quality of life, sleep disruption, social stigma, and high long-term management burden.

Barrier impairment is a central feature of AD. Reduced filaggrin function and downstream changes in stratum corneum organization increase transepidermal water loss (TEWL) and permit greater penetration of irritants and allergens [3]. These defects amplify inflammation, pruritus, and scratching, creating a self-perpetuating itch–scratch cycle. As a result, modern AD management places strong emphasis on basic skin care and regular moisturization to restore barrier function, reduce flare frequency, and improve tolerance of anti-inflammatory medications [4,5,6].

Facial AD presents unique challenges. Even mild erythema, scaling, or excoriation is highly visible and can be psychologically distressing. In addition, periorificial areas and thin facial skin are more susceptible to treatment-related adverse events. Although topical corticosteroids remain effective for acute inflammation, clinicians often limit potency and duration on the face to reduce risks such as epidermal/dermal atrophy, telangiectasia, steroid acne, and perioral dermatitis [5,6]. Steroid-sparing or steroid-limiting options include topical calcineurin inhibitors and proactive maintenance regimens; the cited adult topical-therapy guideline also addresses phosphodiesterase-4 and Janus kinase inhibitors for appropriately selected patients [4,5,6].

Given these considerations, patients frequently seek adjunctive topical products that can be applied safely to the face and that meaningfully improve skin comfort and appearance. Film-forming, water-retentive biomaterials are attractive candidates because they may reduce evaporative loss and provide a physical buffer against irritants. Chitosan is a cationic polysaccharide derived from chitin via deacetylation. It is widely studied in biomedical and cosmetic contexts because it is biocompatible, biodegradable, and capable of forming flexible films on biological surfaces [7,8,9]. Chitosan chemistry varies (degree of deacetylation, molecular weight, charge density, and chemical modification), and these parameters influence solubility, viscosity, bioadhesion, antimicrobial activity, and interactions with skin [8,9].

Several properties make chitosan mechanistically relevant to facial AD. First, chitosan can form a thin, semi-occlusive film that may support hydration, reduce frictional irritation, and decrease sensitivity to environmental triggers [7,8,9]. Second, chitosan and its derivatives have reported antimicrobial effects against selected bacteria—including staphylococci—depending on formulation and conditions [10]. This may matter in AD, where *Staphylococcus aureus* colonization and dysbiosis are common and can exacerbate inflammation through toxin-mediated and immune-activating pathways [11,12]. Third, chitosan-based matrices are frequently discussed as wound-healing supportive materials, potentially relevant to excoriated or fissured atopic skin [13].

Clinical evidence for chitosan-based interventions in AD remains formulation-specific. In a randomized trial enrolling adolescents and adults older than 12 years, severity improved within both the chitosan-coated textile and control-textile groups, without a statistically significant between-group difference in the primary severity outcome [14]. A subsequent randomized trial in children aged 1 to less than 6 years with mild AD found greater improvement with an emulsion containing partially myristoylated carboxymethyl chitosan (PMCMC) than with petrolatum [15]. These interventions cannot be assumed equivalent to the 2% chitosan gel used here in composition or delivery. A review of chitosan-based delivery systems describes hydrogels, films, nanoparticles, and textiles as distinct platforms for anti-inflammatory delivery or barrier support [8].

Despite these signals, there remains limited practical literature describing routine clinical use of topical chitosan gels for facial AD, and standardized image-based outcome documentation is uncommon. Small observational series can still be informative if they use reproducible photography and transparent outcome definitions.

We therefore describe an open-label case series of 20 patients with facial AD treated with a topical 2% chitosan gel (Arche 2% Chitosan, Doum Inc., Seoul, Republic of Korea). To support consistent image interpretation, we introduce a Photographic Atopic Improvement Scale (PAIS) assessed by two independent physicians and present representative before-and-after photographs.

## 2. Materials and Methods

### 2.1. Study Design and Reporting Framework

This was a prospective, open-label case series conducted in an outpatient clinic. The manuscript is structured using observational reporting principles (Strengthening the Reporting of Observational Studies in Epidemiology principles where applicable) with emphasis on transparency of patient selection, intervention protocol, and outcome assessment.

### 2.2. Participants and Eligibility

Consecutive patients presenting with facial AD were screened. Inclusion criteria were (i) a clinical diagnosis of AD with active facial involvement at enrollment; (ii) willingness to follow the standardized skin-care protocol; and (iii) consent for standardized medical photography. Exclusion criteria were (i) active bacterial or viral infection requiring systemic therapy; (ii) known hypersensitivity to chitosan or formulation components; (iii) recent initiation of systemic immunosuppressants or biologics within the prespecified washout period; and (iv) a facial dermatosis considered likely to confound assessment, including uncontrolled rosacea. The original study record available for this report did not document a named diagnostic classification system or a standardized differential-diagnosis checklist for acne, periorificial dermatitis, seborrheic dermatitis, rosacea, or allergic contact dermatitis. This limitation is now stated explicitly and should be considered when interpreting the photographic findings.

### 2.3. Intervention

Participants applied Arche 2% Chitosan gel (Doum Inc., Seoul, Republic of Korea) as a thin layer over affected facial areas twice daily for 4 weeks. All participants were instructed to use a gentle cleanser and a standardized bland moisturizer. New prescription anti-inflammatory agents were discouraged; predefined rescue therapy for flares, including a short course of a low-potency topical corticosteroid or topical calcineurin inhibitor, was permitted and recorded. Two patients used topical tacrolimus during the study. The available study record identifies the investigational product as a 2% chitosan gel but does not provide the chitosan molecular weight, degree of deacetylation, chemical modification, formulation pH, complete excipient composition, vehicle/base, or the specific cleanser and moisturizer product names. The timing, formulation, dosing frequency, and duration of tacrolimus use were also not documented in the dataset available for this report. These unavailable formulation and concomitant-treatment details limit reproducibility and attribution of the observed changes and are acknowledged as study limitations.

### 2.4. Visit Schedule

Assessments were performed at baseline (week 0) and after treatment (week 4). An optional week-8 follow-up had been contemplated to observe durability; however, week-8 completion and outcome data are not available in the dataset used for the present report and therefore were not included in the analysis. The present analysis is restricted to baseline and week-4 outcomes.

### 2.5. Photography Protocol

Standardized facial photographs were captured at baseline and week 4 using a fixed imaging setup with stable lighting, camera-to-subject distance, and reproducible head positioning (chin rest/forehead support when available). Images were taken with a neutral expression and eyes gently closed when needed to reduce motion artifacts and had direct identifying labels removed for analysis. The camera body, lens, exposure settings, light-source specifications, and use of color-calibration targets were not documented in the available study record. Because photographic erythema assessment is sensitive to these factors, the absence of these technical details is acknowledged as a limitation.

### 2.6. Outcome Measures

The available clinical record reports severity assessed using the Eczema Area and Severity Index (EASI), with attention to head/neck involvement, and/or the SCORing Atopic Dermatitis (SCORAD) index, depending on clinic workflow [16,17]. Global clinician severity was recorded using an Investigator Global Assessment (IGA) scale (0 = clear to 4 = severe) [4,5,6]. Patient-reported burden was assessed with the Patient-Oriented Eczema Measure (POEM) and an itch numeric rating scale (NRS; 0–10) [18]. The source label “EASI (head/neck)” is retained in the outcome tables; whether these values represent a total score or a regional calculation could not be established from the available aggregate record. Overall skin quality is used descriptively to refer to visible facial appearance, including erythema and texture, rather than to a prespecified, validated composite endpoint.

### 2.7. Photographic Atopic Improvement Scale (PAIS)

PAIS is a six-point ordinal photographic global-change scale anchored to visible erythema, scale, edema, and inflammatory papulation (Table 1). The scale is unvalidated, and its percentage anchors are approximate visual descriptors rather than measured percentage changes.

Two independent physicians evaluated paired baseline and follow-up images using PAIS. Image pairs were presented in randomized order where feasible, and evaluators were blinded to clinical outcome scores. The available study record does not establish blinding to chronological image order or treatment information. Inter-rater agreement was summarized using weighted kappa.

Before grading, physicians reviewed a brief scoring guide with example images illustrating each PAIS category. For each participant, baseline and follow-up images were displayed side-by-side at identical magnification. Physicians focused on change in (i) erythema intensity and distribution, (ii) degree of scale/roughness, (iii) edema or lichenification, and (iv) inflammatory papulation/excoriation. The protocol contemplated adjudication when physician scores differed by more than one category while preserving the original independent ratings for agreement analysis. However, the number of cases requiring adjudication and the individual score distributions for each rater are not available in the dataset used for this report; consequently, only the available consensus distribution and weighted-kappa estimate are reported.

### 2.8. Exploratory Ultrasound-Based Imaging Record

An additional paired skin ultrasound imaging record was supplied from a SKENA platform (V&CO Inc., Suwon, Republic of Korea). The two saved examinations were dated 13 March and 11 July 2026, 120 days apart. Software-generated indices displayed alongside the imaging assessment were transcribed from the original device dashboard, without conversion to physical units. Acquisition frequency, gain, depth calibration, probe pressure, exact site registration, software version, and index-validation data were not available. Consequently, no dermal-thickness, collagen-content, elasticity, or hydration measurements were derived from the scan colors or proprietary labels. This additional record was not included in the 20-patient baseline/week-4 analyses because its linkage to that cohort and treatment schedule could not be verified. The replacement composite presents two scan panels and three selected device indices. It is reproduced as supplied, without further alteration; additional numerical annotations that could not be reconciled with the original dashboard were not used as outcome measurements.

### 2.9. Statistical Analysis

This observational series was not powered for confirmatory inference. Available baseline and week-4 summary statistics and reported paired comparisons are presented descriptively. The supplied aggregate record did not specify the statistical tests, the dispersion measure accompanying the means, or outcome-specific missingness. Reported *p* values are therefore reproduced without independent recalculation or a claim that the tests were verified. The weighted-kappa estimate and its 95% confidence interval (CI) are retained as provided. No unreported effect estimates, individual rater scores, missing observations, or confidence intervals were reconstructed. The additional ultrasound-based imaging record was assessed descriptively and was not pooled with the cohort data.

### 2.10. Ethics

The 20-patient study was conducted in accordance with the Declaration of Helsinki. Written informed consent for treatment and medical photography was obtained, as documented in the source manuscript. The approval details are given in the Institutional Review Board Statement. Inclusion of the additional imaging record requires separate confirmation of its coverage by the relevant ethics approval and consent for publication.

## 3. Results

### 3.1. Cohort Description

Twenty patients with facial AD were included in the reported cohort. Baseline demographic and clinical characteristics are summarized in Table 2. The week-4 outcome table reports results for the 20-patient cohort; the source material available for this manuscript does not provide a separate missing-data log or outcome-specific missingness record. Accordingly, no unreported imputation or missing-data handling procedure is claimed.

### 3.2. Participant Flow

Participant flow: Consecutive patients were screened, and 20 patients were enrolled in the reported cohort. The source material available for this manuscript does not record the total number screened or excluded, reasons for screening exclusion, or a separate week-8 completion count. The reported baseline and week-4 analyses concern the 20-patient cohort. No week-8 outcomes are included in the present analysis.

### 3.3. Photographic Response

Representative paired clinical photographs are shown in Figure 1, Figure 2 and Figure 3. The photographic ratings suggest improvement in overall facial skin appearance across much of the cohort, while residual erythema and visible lesions remain in some follow-up images. Clinical photographs alone do not establish the diagnosis of AD or reliably distinguish it from overlapping facial dermatoses. Lighting, image acquisition, and physiologic flushing also affect the appearance of erythema and should be considered when comparing the pairs.

### 3.4. PAIS Grading

Two physicians assigned PAIS scores to paired images. The available consensus PAIS distribution was: worse, 0/20 (0%); no change, 1/20 (5%); mild improvement, 5/20 (25%); moderate improvement, 8/20 (40%); marked improvement, 5/20 (25%); and almost clear/clear, 1/20 (5%). Inter-rater agreement was summarized by a weighted kappa of 0.72 (95% CI, 0.55–0.89). Individual rater score distributions and the number of adjudicated cases were not available in the dataset used for this report and therefore cannot be reported without reconstruction of unavailable data.

### 3.5. Clinician- and Patient-Reported Outcomes

Baseline and week-4 clinician- and patient-reported outcomes are summarized in Table 3. The reported SCORAD, POEM, and itch NRS values were lower at week 4, and the IGA distribution shifted toward lower severity categories. The EASI-labeled values also decreased, but the regional-versus-total score definition requires clarification. These findings are compatible with improvement in overall facial skin quality during the chitosan-containing regimen, without establishing a treatment-specific effect. Concomitant skin care, rescue tacrolimus in two participants, and the absence of a control group limit attribution.

### 3.6. Safety and Tolerability

Mild stinging was reported in 3 of 20 patients (15%) and resolved spontaneously. No serious adverse events were recorded in the reported dataset. Because tolerability outcomes were collected in a small uncontrolled cohort, these observations should be considered descriptive.

### 3.7. Additional Ultrasound-Based Imaging Findings

The additional paired imaging record is presented in Figure 4, which displays three selected software-generated indices. In the original device dashboard, Booster Regen changed from 43 to 56, Booster Moist from 35 to 48, and Collagen Tightening from 46 to 53 between 13 March and 11 July 2026. The corresponding absolute changes were +13, +13, and +7 displayed score units. The same source also recorded increases in Lift (24 to 55), Toning (51 to 61), and Hybrid (41 to 59), which are not separately shown in the replacement composite. The replacement graphic contains additional rightmost values of 61, 59, and 61 in its after-treatment panel; these are not reconciled with the original dashboard and were not treated as additional measurements. The score definitions, direction of clinical benefit, and measurement properties were not supplied, so these numerical changes cannot independently establish improved hydration, collagen remodeling, lifting, or pigmentation. The examinations were 120 days apart and are not designated as baseline/week-4 observations. No cohort-level effect estimate or significance test was calculated from this single paired record.

## 4. Discussion

This small, uncontrolled, open-label case series associates a topical 2% chitosan-containing skin-care regimen with improvement in overall facial skin appearance and available clinical outcomes. It also illustrates a pragmatic photographic improvement scale. These changes cannot be attributed solely to chitosan because there was no vehicle or moisturizer control, AD fluctuates naturally, moisturizer use was concomitant, and two patients received rescue topical tacrolimus. Accordingly, the findings are hypothesis-generating rather than confirmatory evidence of efficacy.

### 4.1. How Chitosan Could Influence Facial AD

AD pathogenesis involves barrier dysfunction and inflammation amplified by microbial dysbiosis [1,2]. Chitosan gels may address multiple components of this loop. Film formation may reduce evaporative water loss and mechanical irritation, consistent with guideline emphasis on moisturization as a core intervention [4,5,6]. In addition, chitosan’s cationic nature can facilitate electrostatic interactions with negatively charged skin surfaces and proteins, potentially increasing residence time and reducing friction from masks or environmental exposure in the facial region [7,8,9].

### 4.2. Microbiome Considerations

*S. aureus* colonization is common in AD and can worsen inflammation through toxins, superantigens, and barrier-disrupting enzymes [11,12]. Narrative reviews emphasize that strategies targeting dysbiosis—while avoiding unnecessary topical antibiotics—remain an area of active research [11]. Some chitosan-based materials demonstrate antimicrobial effects against staphylococci in vitro and in applied contexts (e.g., impregnation in textiles), although the magnitude and clinical relevance depend strongly on formulation, concentration, and local conditions such as pH [10]. A chitosan gel used on facial AD might therefore contribute to symptomatic improvement by simultaneously supporting hydration and creating an unfavorable microenvironment for bacterial overgrowth, but direct microbiome measurement would be needed to substantiate this mechanism.

### 4.3. Consistency with Clinical Evidence

The textile and emulsion trials should not be treated as interchangeable evidence for topical chitosan gel. The textile trial enrolled adolescents and adults and did not demonstrate superiority over control textiles for the primary severity outcome [14]. The PMCMC-containing emulsion trial reported greater improvement than petrolatum in young children with mild AD [15]. Neither establishes efficacy for the formulation used in the present facial case series. Differences in molecular characteristics, vehicle, population, and delivery route may determine whether a chitosan-based product acts primarily as a film-forming moisturizer, delivery matrix, or antimicrobial adjunct [8,9].

### 4.4. Value and Limitations of PAIS

Standardized photography is increasingly used in dermatology but interpretation can vary across clinicians. PAIS is designed as a simple ordinal tool for paired image grading, similar in spirit to global improvement scales in aesthetic medicine. Its strengths include ease of use and direct linkage to visible patient-relevant signs (redness, papulation). However, PAIS requires formal validation for reliability and sensitivity to change. Future studies should assess inter- and intra-rater reliability and correlate PAIS with validated severity scores such as EASI/SCORAD and POEM [16,17,18].

### 4.5. Safety in Facial Application

Facial skin can react to topical products with irritant dermatitis, acneiform eruptions, or periorificial dermatitis. Chitosan is widely regarded as biocompatible, but allergy or intolerance to formulation components may occur. Because some AD patients have heightened sensory symptoms (stinging and burning), tolerability should be measured explicitly in future trials. These concerns reinforce the distinction between general material biocompatibility and the tolerability of a particular topical preparation [7,8,9].

### 4.6. Limitations

The major limitations are the lack of a vehicle/moisturizer control, small cohort size, open-label design, and potential confounding from concurrent moisturizer use and rescue topical tacrolimus in two participants. AD fluctuates naturally; regression to the mean and seasonality cannot be excluded. The available study record did not provide a named diagnostic classification system, a standardized differential-diagnosis checklist, detailed chitosan physicochemical characteristics (molecular weight, degree of deacetylation, chemical modification, or pH), complete excipient composition, specific cleanser/moisturizer product names, detailed tacrolimus timing and dosing, individual PAIS rater distributions, the number of adjudicated PAIS cases, or analyzable week-8 outcomes. Photographic endpoints remain sensitive to lighting, camera settings, color calibration, and physiologic flushing; detailed camera/lens/exposure/light-source specifications and color-calibration information were not available. Post-treatment hyperpigmentation was not a prespecified outcome and was not systematically quantified; therefore, no conclusion regarding its incidence can be drawn from this series. Finally, ethnicity was not reported as an analyzed cohort characteristic, and this manuscript does not infer participant ethnicity from facial photographs. The report also lacks microbiome assessment, barrier-function testing (e.g., TEWL), and biomarker measurements.

The separately presented imaging record has additional limitations: it is a single pair of examinations 120 days apart, its relationship to the study cohort and chitosan exposure is unconfirmed, and concomitant procedures or products cannot be excluded from the available record. Proprietary score definitions and acquisition parameters were unavailable. The images therefore cannot validate an ultrasound biomarker of AD improvement or substantiate treatment-specific effects on overall skin quality. The EASI label and the statistical methods underlying the reported aggregate results also require clarification. The replacement composite also contains numerical annotations that are inconsistent with the original dashboard; those annotations were excluded from the descriptive outcome interpretation.

### 4.7. Future Directions

A randomized, vehicle-controlled trial would allow estimation of effect size and separation of chitosan-specific effects from moisturizing vehicle effects. Such a trial could incorporate validated outcomes, tolerability, and objective barrier metrics, as well as microbiome sequencing to test hypotheses about *S. aureus* suppression. Phenotype stratification (erythematous vs. lichenified facial AD, presence of periorificial involvement, and baseline colonization status) may help identify responders.

### 4.8. Formulation and Translational Considerations

Chitosan is not a single, uniform ingredient. Its behavior depends on degree of deacetylation, molecular weight, charge density, chemical modification, vehicle pH, and excipients [7,8,9]. Solubilizing acids or salts may influence both physical properties and tolerability on compromised skin. Product-specific physicochemical characterization is therefore necessary before extrapolating findings between formulations. The clinical PMCMC trial tested a chemically modified chitosan-containing emulsion, not the uncharacterized 2% gel used here [15]. A nominal chitosan concentration alone does not establish comparable barrier interactions or biological activity.

### 4.9. Differential Diagnosis and Lesion Interpretation

Facial dermatitis in real-world practice frequently overlaps with acne, rosacea, seborrheic dermatitis, or periorificial dermatitis, and these conditions can alter the photographic appearance of papules and erythema. When using a photographic endpoint, clinicians should document key diagnostic features (distribution, scale quality, pruritus, response history) and consider dermoscopic or microbiologic assessment when uncertainty exists. This is particularly relevant because semi-occlusive films may improve xerosis yet, in predisposed individuals, can exacerbate follicular occlusion and acneiform eruptions. Future trials should predefine acneiform adverse-event monitoring and consider excluding patients with uncontrolled acne or rosacea [1,2,7,8,9].

### 4.10. Outcome Measurement Strategy

Validated instruments such as EASI, SCORAD, and POEM facilitate standardized assessment when administered and reported according to their original definitions [16,17,18]. Any regional adaptation should be distinguished explicitly from the validated total score. Standardized photography and PAIS may provide complementary descriptive information, but PAIS requires validation. Future objective assessments should document acquisition settings and repeatability, and use validated erythema, hydration, barrier-function, or ultrasound measures rather than infer biological changes from proprietary treatment-oriented labels.

### 4.11. Patient Adherence and Acceptability

In AD, daily adherence to topical regimens is a key determinant of outcome and is influenced by sensory attributes (greasiness, stickiness, stinging), time burden, and perceived benefit. Film-forming gels may be preferred by some patients because they absorb quickly and create a ‘protective layer,’ but they may feel tight or tacky for others. These formulation and adherence considerations are consistent with the emphasis on tolerable basic skin care in the cited guidelines [4,5,6]. Capturing adherence (e.g., patient diary, product weight change) and patient satisfaction would add interpretive value in future chitosan studies.

### 4.12. Interpreting Overall Skin Quality and Device-Based Indices

The additional imaging record illustrates how ultrasound and software-generated indices might supplement future assessments of overall skin quality. The numerical increases in the original dashboard are descriptive observations, not proof of regeneration or a direct chitosan effect. In particular, labels such as “Collagen Tightening” and “Lift” are not equivalent to measured collagen synthesis, biomechanical tightening, or anatomical displacement. The embedded efficacy statement in the author-supplied composite is not treated as a validated study conclusion, and its unreconciled numerical annotations were not analyzed. Establishing physiological or treatment-specific effects would require validated endpoints, a reproducible acquisition protocol, appropriate controls, and confirmation of treatment timing and cohort eligibility. The clinical findings are therefore most accurately described as an association between the chitosan-containing regimen and improved overall facial appearance, while the additional device record remains exploratory.

Taken together, these considerations support the view that topical chitosan gels are best studied as formulation-specific, barrier-supportive adjuncts. Their role may be to improve comfort and visible redness while reducing reliance on higher-potency anti-inflammatory agents on the face, but robust controlled evidence is needed.

## 5. Conclusions

In this small, uncontrolled observational series, topical 2% chitosan gel was feasible and generally well tolerated, with mild transient stinging in 3 of 20 patients. The chitosan-containing regimen was associated with improvement in overall facial skin quality as reflected by available clinical and photographic assessments over 4 weeks, but this descriptive association does not establish treatment-specific efficacy. PAIS requires validation, and the separately presented ultrasound-based record does not provide an independently validated measure of improvement. Controlled studies with fully characterized formulations, verified outcome definitions, and standardized imaging are needed.

## Figures and Tables

**Figure 1:**
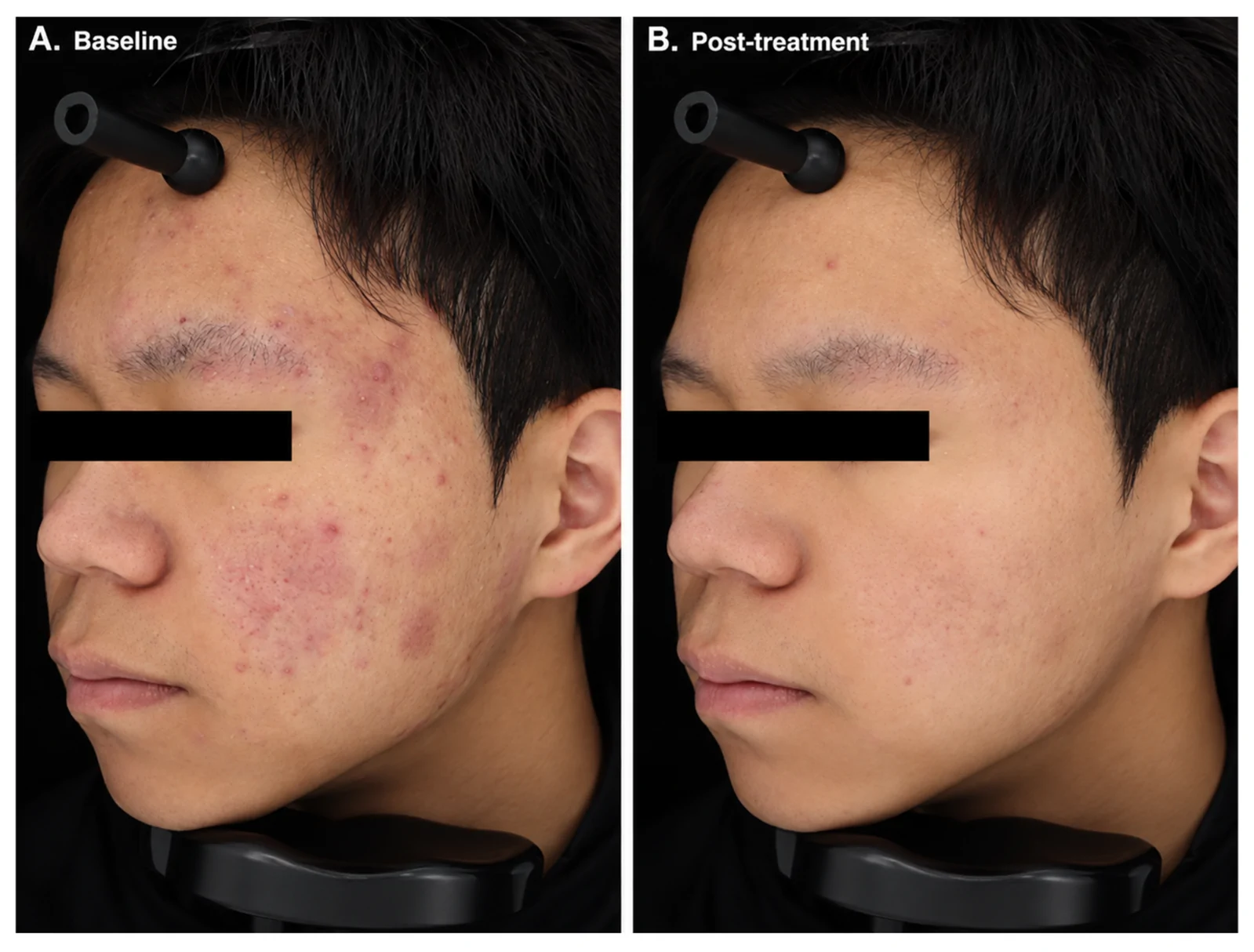
Lateral facial photographs at baseline (**A**) and the week-4 assessment after topical 2% chitosan gel use (**B**) in a representative patient with clinically diagnosed facial atopic dermatitis. The pair illustrates changes in visible erythema and papular lesions across the forehead, temple, and cheek. The Photographic Atopic Improvement Scale (PAIS) was used for cohort assessment; this photographic comparison is descriptive and is not a quantitatively color-calibrated measure of inflammation.

**Figure 2:**
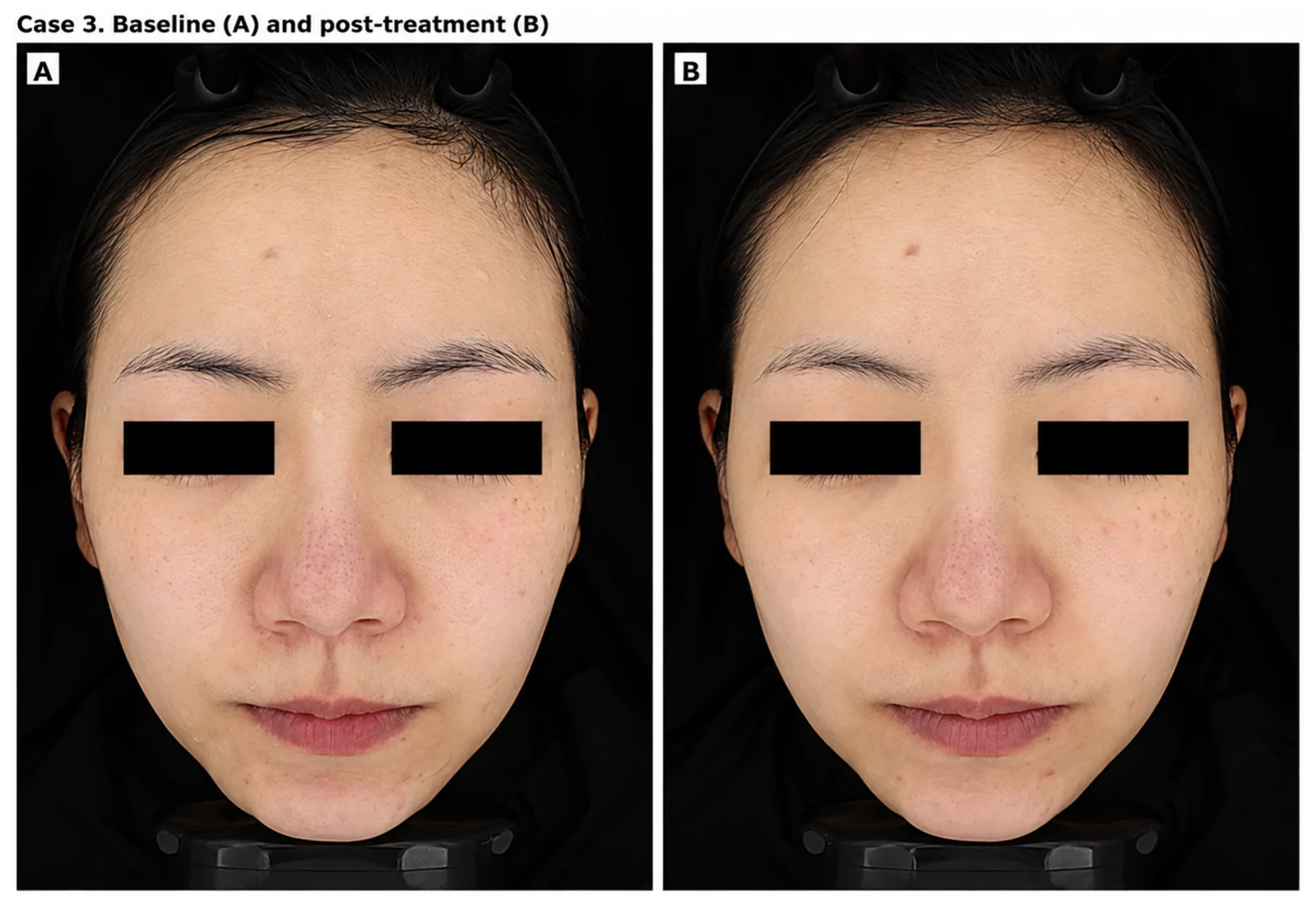
Frontal facial photographs obtained before treatment (**A**) and after 4 weeks of topical 2% chitosan gel use (**B**) in a further representative case of clinically diagnosed facial atopic dermatitis. The images permit comparison of erythema distribution and visible surface irregularity across the central face. The Photographic Atopic Improvement Scale (PAIS) was used for cohort assessment; apparent change should be interpreted in view of the unreported color-calibration and detailed acquisition parameters.

**Figure 3:**
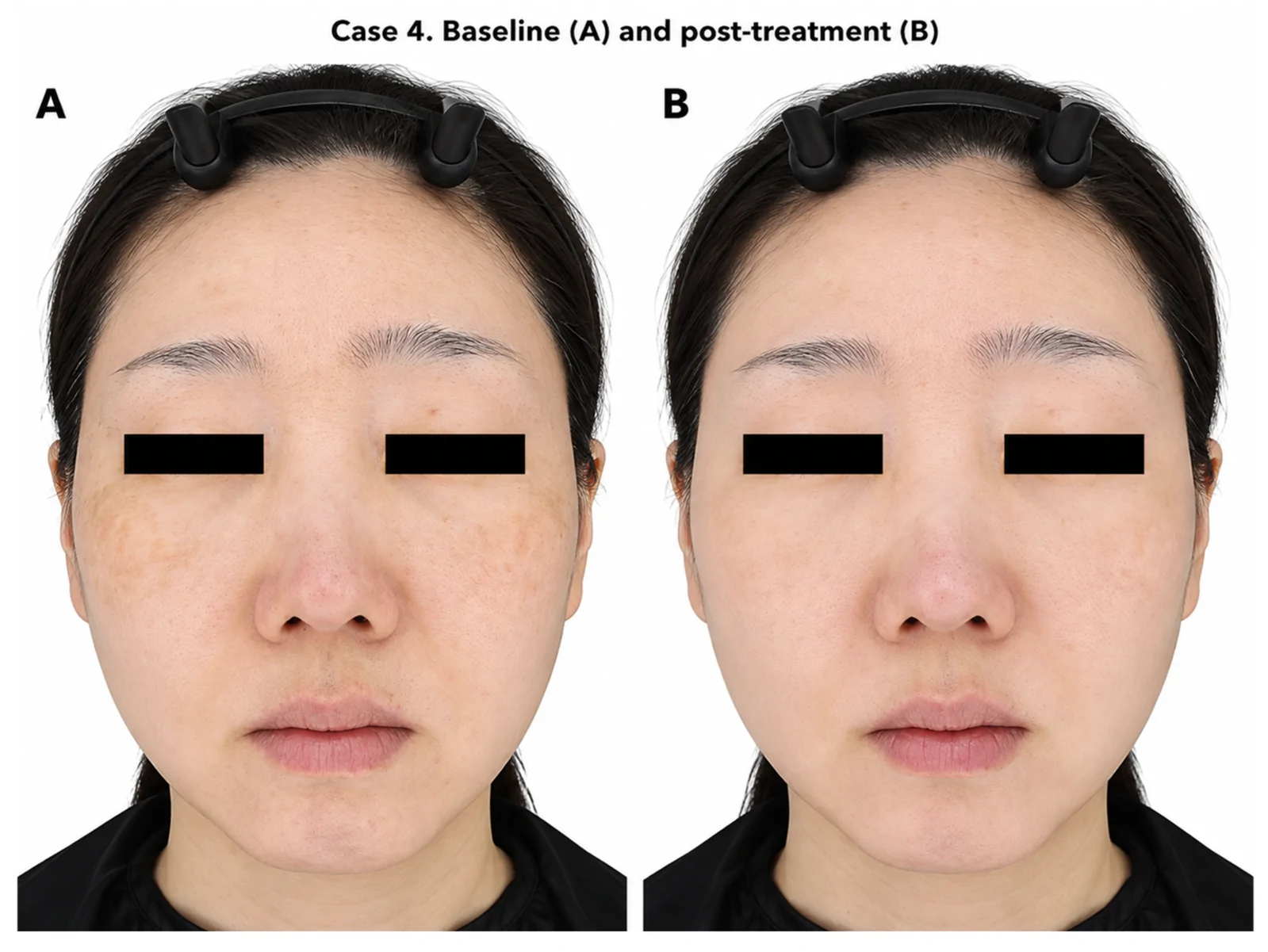
Additional paired frontal photographs showing baseline facial appearance (**A**) and the week-4 assessment following topical 2% chitosan gel use (**B**). The pair illustrates overall facial appearance, including residual erythema and textural variation, rather than a direct measurement of skin-barrier function. The Photographic Atopic Improvement Scale (PAIS) was used for cohort assessment; these descriptive images do not establish that chitosan alone caused the observed changes.

**Figure 4:**
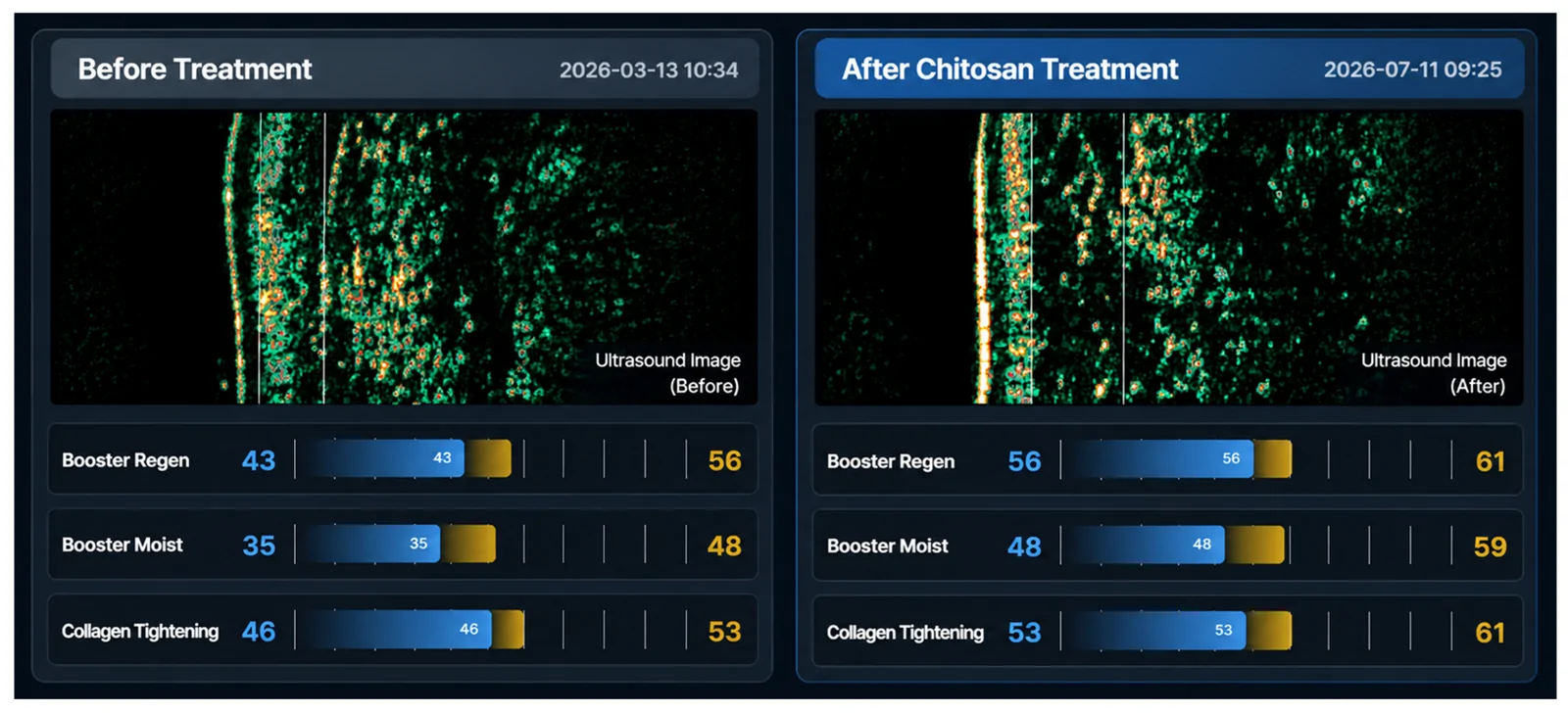
Author-supplied composite of paired ultrasound-based skin imaging and selected device-displayed indices from SKENA (V&CO Inc., Republic of Korea). The left and right scan panels are labeled before treatment (13 March 2026) and after treatment (11 July 2026), respectively, an interval of 120 days rather than the cohort’s four-week assessment interval. The original dashboard records Booster Regen as 43 to 56, Booster Moist as 35 to 48, and Collagen Tightening as 46 to 53. Additional rightmost values of 61, 59, and 61 in the supplied after-treatment panel are not reconciled with that source and are not used as outcomes. The composite and its embedded annotations are reproduced without alteration; the proprietary labels and embedded efficacy statement do not establish direct measurements of regeneration, hydration, collagen synthesis, or treatment-specific efficacy. This additional record was not included in the 20-patient cohort analysis.

**Table 1:** Photographic Atopic Improvement Scale (PAIS): scoring anchors.

Score	Descriptor	Operational Definition (Photographic Signs)
0	Worse	Increase in erythema, scale, edema, crusting, or papulation compared with baseline.
1	No change	No meaningful visible change (≤10–20% change) in dominant facial signs.
2	Mild improvement	Small but visible reduction in erythema and/or scale and/or papulation (approximately 25% improvement).
3	Moderate improvement	Clear reduction in erythema and inflammatory signs (approximately 50% improvement).
4	Marked improvement	Near-complete resolution of visible signs with only faint residual erythema or texture change (approximately 75% improvement).
5	Almost clear/clear	Minimal to no visible active dermatitis; residual post-inflammatory color change may remain.

Percentage anchors are approximate visual estimates, not quantitative measurements. PAIS has not undergone formal validation.

**Table 2:** Baseline characteristics of the cohort (*n* = 20).

Characteristic	Value
Age, years	32.5 ± 8.7 (18–55)
Sex, *n* (%)	Female: 14 (70%)/Male: 6 (30%)
AD duration, years	6.2 ± 4.8
Baseline EASI (reported as head/neck) *	7.5 ± 2.8
Baseline SCORAD	38.4 ± 10.2
Baseline IGA, *n* (%)	Moderate (3): 20 (100%)
Baseline POEM	18.2 ± 3.5
Baseline itch NRS (0–10)	6.8 ± 1.6
Concomitant moisturizers	Standardized bland moisturizer permitted
Rescue anti-inflammatory therapy	Permitted under predefined flare criteria; recorded (2 patients used topical tacrolimus)

AD, atopic dermatitis; EASI, Eczema Area and Severity Index; SCORAD, SCORing Atopic Dermatitis; IGA, Investigator Global Assessment; POEM, Patient-Oriented Eczema Measure; NRS, numeric rating scale. Summary values are reproduced as supplied; the meaning of the dispersion term requires confirmation. * The source EASI label is retained, but the regional-versus-total score definition was not available.

**Table 3:** Baseline and week-4 clinical, patient-reported, photographic, and safety outcomes (*n* = 20).

Outcome	Baseline	Week 4	Reported Change/Statistics
EASI (reported as head/neck) *	7.5 ± 2.8	2.7 ± 1.9	−4.8 ± 1.9 (*p* < 0.001)
SCORAD	38.4 ± 10.2	14.8 ± 8.1	−23.6 ± 8.5 (*p* < 0.001)
IGA, *n* (%) by category			Reported category shift: *p* < 0.001 †
Clear (0)	0 (0%)	4 (20%)	
Almost clear (1)	0 (0%)	5 (25%)	
Mild (2)	0 (0%)	7 (35%)	
Moderate (3)	20 (100%)	4 (20%)	
Severe (4)	0 (0%)	0 (0%)	
POEM	18.2 ± 3.5	8.3 ± 4.1	−9.9 ± 3.2 (*p* < 0.001)
Itch NRS (0–10)	6.8 ± 1.6	2.4 ± 1.8	−4.4 ± 1.7 (*p* < 0.001)
PAIS consensus score distribution, *n* (%)	N/A		Weighted κ = 0.72 (95% CI, 0.55–0.89)
Worse (0)		0 (0%)	
No change (1)		1 (5%)	
Mild improvement (2)		5 (25%)	
Moderate improvement (3)		8 (40%)	
Marked improvement (4)		5 (25%)	
Almost clear/clear (5)		1 (5%)	
Adverse events, *n* (%)	N/A	Mild stinging: 3 (15%)	All resolved spontaneously.

EASI, Eczema Area and Severity Index; SCORAD, SCORing Atopic Dermatitis; IGA, Investigator Global Assessment; POEM, Patient-Oriented Eczema Measure; NRS, numeric rating scale; PAIS, Photographic Atopic Improvement Scale; CI, confidence interval; N/A, not applicable. Values and *p* values are reproduced from the aggregate source record, not recalculated; the statistical tests, dispersion measure, and outcome-specific denominators require confirmation. * The regional-versus-total EASI definition is unresolved. † The method used to test the IGA category shift was not specified.

## Data Availability

The available aggregate results and representative images are presented in this article. Requests concerning the underlying study records should be directed to the corresponding author and remain subject to applicable consent and privacy requirements. Individual-level data and a complete acquisition protocol for the additional imaging record were not supplied for this revision.

## Abbreviations

Abbreviation	Definition
AD	Atopic dermatitis
CI	Confidence interval
EASI	Eczema Area and Severity Index
IGA	Investigator Global Assessment
NRS	Numeric rating scale
PAIS	Photographic Atopic Improvement Scale
PMCMC	Partially myristoylated carboxymethyl chitosan
POEM	Patient-Oriented Eczema Measure
SCORAD	SCORing Atopic Dermatitis
TEWL	Transepidermal water loss
